# On the Long Time Simulation of Reaction-Diffusion Equations with Delay

**DOI:** 10.1155/2014/186802

**Published:** 2014-02-03

**Authors:** Dongfang Li, Chengjian Zhang

**Affiliations:** School of Mathematics and Statistics, Huazhong University of Science and Technology, Wuhan 430074, China

## Abstract

For a consistent numerical method to be practically useful, it is widely accepted that it must preserve the asymptotic stability of the original continuous problem. However, in this study, we show that it may lead to unreliable numerical solutions in long time simulation even if a classical numerical method gives a larger stability region than that of the original continuous problem. Some numerical experiments on the reaction-diffusion equations with delay are presented to confirm our findings. Finally, some open problems on the subject are proposed.

## 1. Introduction

Reaction-diffusion equations with delay have attracted a significant interest in the last several decades due to their frequent appearance in actual applications [1–11]. They are usually proposed as models for neural networks, population ecology, and cell biology. As far as we know, a delay term could not only make the exact solutions difficult to be obtained in general but also change the long time dynamic properties of a system. That is the reason why such equations became the focus of researchers in numerical analysis and simulation. Up to now, most of the numerical methods available to approximate the diffusion term are based on some classical numerical methods, for example, central finite difference method [3–7], compact finite difference method [8, 9], discontinuous Galerkin methods [10], spectral collocation method [11], and so forth. Many important properties of these numerical methods such as asymptotic stability and convergence have been extensively investigated. However, little research has been conducted in studying the long time dynamical properties of these numerical methods.

The long time dynamical properties of different numerical methods for PDEs are attractive (e.g., [12, 13]). As a typical example in the delay field, we refer the readers to the works [14, 15], where the authors considered the following reaction-diffusion equation with delay:

(1) $$\begin{array}{c} \frac{\partial u}{\partial t}\left(x,t\right)=a\frac{\partial^{2}u}{\partial x^{2}}\left(x,t\right)+bu\left(x,t-\tau \right),\;x\in \left(0,\pi \right),\;\;t>0,\\ u\left(0,t\right)=u\left(\pi ,t\right)=0,\;t>0,\\ u\left(x,t\right)=\varphi \left(x,t\right),\;\left(x,t\right)\in \left[0,\pi \right]\times \left[-\tau ,0\right]. \end{array}$$

As it is pointed out in the papers [14, 15], the spatial discretization based on standard central difference method (CDM) unavoidably leads to a reduction of the asymptotic stability region of the corresponding PDE problem. In other words, the long time numerical solutions derived by CDM may be unstable even if the original problem (1) is stable. As a result, it is widely accepted that a numerical method should preserve stability of the original continuous problems in actual application (e.g., [14–20]). This naturally leads to the following question: are the long time numerical solutions reliable if the discrete problem has a larger stability region than that of the continuous one?

In this study, it is shown that a negative answer to the above question can be obtained. The conclusion is obtained when we try to find some high-order numerical methods, which are sufficient to preserve stability of the continuous problem. It is well known that the finite element method (FEM) can be easily designed for high order of accuracy in space. We have proved that one-degree FEM gives a larger stability region than that of the continuous problem. It means that the numerical method is sufficient to preserve the asymptotic stability of the original problems. Numerical experiments indicate that the long time numerical solutions decay in time. However, after some more numerical tests, we find that the long time numerical solutions derived by FEM may not be reliable for the following two reasons. First, the numerical solutions derived by the FEM decay more quickly than the ones by the alternative central difference method (ACDM). The latter method is proved to be a good choice for solving the problem (1) in [15]. Second, if we reduce the stepsize, the long time FEM solutions at every fixed time have changed, while the solutions derived by ACDM nearly remain the same.

The rest of the paper is organized as follows.  Section 2 describes in detail the FEM and stability analysis of the problem.  Section 3 shows experimental studies for verifying the proposed results. Finally, conclusions and discussions for this paper are summarized in Section 4.

## 2. Main Results

In this section, after a statement of the classical FEM, stability of the discrete reaction diffusion equations with delay is investigated.

For the spatial discretization of system (1), we divide the interval *I* = [0, *π*] with a mesh: 0 = *x*_0_ < *x*_1_ < ⋯<*x*_*N*_ = *π* with the space stepsize *h* = *π*/*N*. Let *S* ^*h*^ be a finite dimensional space; then the semidiscrete FEM is to find *U* ∈ *S* ^*h*^, such that for all test functions *χ*_*i*_ ∈ *S* ^*h*^ and 1 ≤ *i* ≤ *N*, satisfying

(2) ∫I∂U∂t(x,t)χi dx+a∫I∂U∂x∂χi∂x dx  =b∫IU(x,t−τ)χi dx, t>0.

For the classical one-degree FEM, the piecewise linear function *χ*_*k*_ is chosen as follows: *χ*_0_(*x*) = (*x*_1_ − *x*)/(*x*_1_ − *x*_0_), *χ*_*N*_(*x*) = (*x* − *x*_*N*−1_)/(*x*_*N*_ − *x*_*N*−1_), and

(3) $$\begin{array}{c} \chi_{i}\left(x\right)=\{\begin{array}{cc} \frac{x-x_{i-1}}{h}, & x\in \left[x_{i-1},x_{i}\right],\\ \frac{x_{i+1}-x}{h}, & x\in \left[x_{i},x_{i+1}\right],\\ 0, & \text{otherwise}, \end{array}\;0<i<N. \end{array}$$

Let *U* = ∑_0_^*N*^ *u*_*i*_(*t*)*χ*_*i*_(*x*); one can derive the following delay differential equations in the matrix form:

(4) $$\begin{array}{c} BU^{\prime }=aAU+bBU\left(t-\tau \right). \end{array}$$

Here *U* = (*u*_1_(*t*), *u*_2_(*t*),…, *u*_*N*−1_(*t*)) with *u*_*i*_(*t*) denoting an approximation to *u*(*ih*, *t*), *B* = (*h*/6)(4*I* + *S*), and *A* = (1/*h*)(−2*I* + *S*), where

(5) $$\begin{array}{c} S={\left[\begin{array}{ccccc} 0 & 1 & \cdots & 0 & 0\\ 1 & 0 & \cdots & 0 & 0\\ \vdots & \vdots & \vdots & \vdots & \vdots \\ 0 & 0 & \cdots & 0 & 1\\ 0 & 0 & \cdots & 1 & 0 \end{array}\right]}_{\left(N-1)\times (N-1\right)} \end{array}$$

with its eigenvalues *λ*_*j*_^*S*^ = 2cos⁡ (*jπ*/*N*), *j* = 1,2,…, *N* − 1.

In what follows, we will turn our attention to the stability analysis of the semidiscrete PDE (4). First, we introduce some lemmas, which will assist in the proof of our main result.

**Lemma 1**

Suppose that *x* is an eigenvector of the matrix *S* with the corresponding eigenvalue *λ*_*j*_^*s*^. Then, *x* is also an eigenvector of the matrix *B* ^−1^ *A* with the corresponding eigenvalue *λ*_*j*_^*B*^−1^*A*^ = 6(−2 + *λ*_*j*_^*S*^)/*h* ^2^(4 + *λ*_*j*_^*S*^), where matrixes *A* and *B* are defined in (4). Moreover, the maximum eigenvalue of the matrix *B* ^−1^ *A* is given by

(6) $$\begin{array}{c} \lambda_{1}^{B^{-1}A}=\frac{6\left(-2+2\cos\left(h\right)\right)}{h^{2}\left(4+2\cos\left(h\right)\right)}<-1. \end{array}$$

**Proof**

It follows from *Sx* = *λ*_*j*_^*s*^ *x* that

(7) $$\begin{array}{c} Ax=\frac{1}{h^{2}}\left(-2I+S\right)x=\frac{1}{h^{2}}\left(-2+\lambda_{j}^{S}\right)x,\\ Bx=\frac{1}{6}\left(4I+S\right)x=\frac{1}{6}\left(4+\lambda_{j}^{S}\right)x. \end{array}$$

Hence,

(8) $$\begin{array}{c} Ax=\frac{1}{h^{2}}\left(-2+\lambda_{j}^{S}\right)x=\frac{1}{h^{2}}\left(-2+\lambda_{j}^{S}\right)\cdot \frac{6}{\left(4+\lambda_{j}^{S}\right)}Bx. \end{array}$$

Therefore,

(9) $$\begin{array}{c} B^{-1}Ax=\frac{6\left(-2+\lambda_{j}^{S}\right)}{h^{2}\left(4+\lambda_{j}^{S}\right)}x. \end{array}$$

Meanwhile, noting that *h* = *π*/*N*, we have

(10) λjB−1A=6(−2+2cos⁡(jπ/N))h2(4+2cos⁡(jπ/N))=6(−2+2cos⁡(jh))h2(4+2cos⁡(jh))=−24 sin2(jh/2)h2(4+2cos⁡(jh)), j=1,2,…,N−1.

When *j* = 1, we have the maximum denominator *h* ^2^(4 + 2cos⁡ (*h*)) and the minimum numerator 24 sin^2^ (*h*/2). Thus, the maximum eigenvalue is

(11) $$\begin{array}{c} \lambda_{1}^{B^{-1}A}=\frac{6\left(-2+2\cos\left(h\right)\right)}{h^{2}\left(4+2\cos\left(h\right)\right)}. \end{array}$$

Moreover, substituting the Maclaurin series expansions

(12) $$\begin{array}{c} \cos\left(h\right)=1-\frac{h^{2}}{2}+\frac{h^{4}}{24}+\cdots \end{array}$$

into (11) yields

(13) $$\begin{array}{c} \lambda_{1}^{B^{-1}A}=-\frac{12-h^{2}+𝒪\left(h^{4}\right)}{12-2h^{2}+𝒪\left(h^{4}\right)}<-1, \end{array}$$

which completes the proof.

**Lemma 2 (cf. [14])**

Suppose that all roots of the following equation:

(14) $$\begin{array}{c} \lambda =\alpha +\beta \exp\left(-\lambda \tau \right), \end{array}$$

have negative real part for any given *α* ≤ 0, *β* ∈ ℝ. Then for any *x* ≤ *α*, all the roots *λ* of the equation

(15) $$\begin{array}{c} \lambda =x+\beta \exp\left(-\lambda \tau \right) \end{array}$$

have negative real part too.

The following lemma can be derived from [14, Corollary  2.12] or [16, Proposition  1].

**Lemma 3**

The following equation:

(16) $$\begin{array}{c} \lambda =\alpha +\beta \exp\left(-\lambda \tau \right), \end{array}$$

has negative real part if and only if *ατ* < 1 and *α* < −*β* < *θ*/*τ*sin*θ*, where *θ* is the root of *θ*cos⁡*θ* = −*ατ*sin*θ* such that 0 < *θ* < *π*.

**Theorem 4**

Assume *a* ≥ 0. Then the zero solution of the semidiscrete delay PDE (2) is asymptotically stable if and only if *aλ*_1_^*B*^−1^*A*^ < −*b* < *θ*/*τ*sin*θ*, where *λ*_1_^*B*^−1^*A*^ is defined in (6) and *θ* is the root of *θ*cos⁡*θ* = −*aτ*sin*θ* such that 0 < *θ* < *π*.

**Proof**

The zero solution to semidiscrete delay PDE (4) is asymptotically stable if all the roots of the corresponding characteristic equation

(17) $$\begin{array}{c} \det\left(\left(\lambda -b\exp\left(-\lambda \tau \right)\right)I_{N-1}-aB^{-1}A\right)=0 \end{array}$$

have negative real parts.

According to Lemma 1, the eigenvalues of the matrix *B* ^−1^ *A* are *λ*_*j*_^*B*^−1^*A*^. Therefore, the asymptotic stability of system is equivalent to the condition that all the roots of each of the equations

(18) $$\begin{array}{c} \lambda -b\exp\left(-\lambda \tau \right)-a\lambda_{j}^{B^{-1}A}=0 \end{array}$$

have the negative real part.

Together with Lemma 2 and the maximum eigenvalue *λ*_1_^*B*^−1^*A*^ (it is proved in Lemma 1), we claim that the asymptotic stability of (2) is equivalent to the condition that the root of the following equation:

(19) $$\begin{array}{c} \lambda -b\exp\left(-\lambda \tau \right)-a\lambda_{1}^{B^{-1}A}=0, \end{array}$$

has the negative real part.

Finally, application of Lemma 3 gives the result.

The asymptotical stability of the original PDE (1) is shown as follows.

**Lemma 5 (cf. [15])**

The zero solution of (1) is asymptotically stable if and only if *a* ≥ 0 and −*a* < −*b* < *θ*/*τ*sin*θ*, where *θ* is the root of *θ*cos⁡*θ* = −*aτ*sin*θ* such that 0 < *θ* < *π*.

The preceding theorem and Lemma 5 immediately yield the following result.

**Theorem 6**

The spatial discretization based on standard FEM gives a larger asymptotic stability region than that of the corresponding PDE.

**Proof**

Together with *λ*_1_^*B*^−1^*A*^ < −1 (it is proved in Lemma 1), Lemma 5, and Theorem 4, we have the conclusion.

Theorem 6 implies that the FEM, combined with an appropriate time discretization, can be sufficient to preserve the asymptotic stability of the continuous delay PDEs (1). However, in the next section, we will show that one-degree FEM may lead to unreliable long time numerical solutions.

## 3. Numerical Experiments

In this section, we will present several numerical experiments to illustrate our findings, where the diffusion term is discreted by the FEM, the CDM

(20) $$\begin{array}{c} \frac{\partial^{2}u}{\partial x^{2}}\approx \frac{u_{i+1,j}-2u_{i,j}+u_{i-1,j}}{h^{2}}, \end{array}$$

and the ACDM (cf. [14, 15])

(21) $$\begin{array}{c} \frac{\partial^{2}u}{\partial x^{2}}\approx \frac{u_{i+1,j}-2u_{i,j}+u_{i-1,j}}{{\left(2\sin(h/2)\right)}^{2}}, \end{array}$$

respectively, where *u*_*i*,*j*_ is a numerical approximation to *u*(*x*_*i*_, *t*_*j*_). Moreover, the time discretization is done by the trapezoidal rule, which is proved as a good choice for long term numerical simulation of (1.1) in [15].

We firstly show that the stability region of the discrete delay PDE derived by FEM is larger than that of the original continuous problem. To do this, we set *τ* = 1, *a* = 1, *b* = 1.01, the time interval [0,1200], and the following initial condition:

(22) $$\begin{array}{c} u_{0}\left(x,t\right)=\exp\left(-2t\right)\sin\left(x\right). \end{array}$$

According to Lemma 1, the original problem is not asymptotically stable. Now, combined with the previously mentioned time discretization, we set space stepsize *h* = *π*/8 and time stepsize *k* = 1/16 and apply the CDM, the ACDM, and the FEM to discrete the equation, respectively. The numerical solutions at different time are listed in Table 1. As it is shown, the numerical solutions derived by finite element method decay in time. It indicates that the stability region of discrete equation derived by FEM is larger than that of the original problem.

Next, we will show that the FEM solutions may not be reliable. Let *τ* = 1, *a* = 1, and *b* = 0.99. It is easy to check that the original delay PDE is asymptotically stable. Now, we still set space stepsize *h* = *π*/8 and time stepsize *k* = 1/20 and apply the different numerical method to solve problem (1) with the initial condition (22). Some statistics of the numerical results are presented in Table 2. Clearly, the numerical solutions derived by the CDM increase in time. And there is a decay of the numerical solutions derived by the other two methods. However, we find that the numerical solutions derived by the FEM decay more quickly than that by the ACDM. In order to distinguish which numerical solutions are reliable, we reduce the stepsize and let space stepsize *h* = *π*/16 and time stepsize *k* = 1/40. The numerical results with different methods can be found in Table 3. It is shown that the numerical solutions derived by ACDM remain the same, while the others have changed, which confirm our findings.

## 4. Conclusions and Discussions

It is pointed out in [14, 15] that the spatial discretization based on standard CDM unavoidably leads to a reduction of the asymptotic stability region of the corresponding continuous PDE problem. Therefore, for a numerical method to be practically useful for the long time simulation, it must preserve the asymptotic stability of the original continuous problem. It is reasonable but not enough. In this study, it is shown that the FEM is sufficient to preserve the asymptotically stability of the corresponding PDE problem, but the method may give long time unreliable solutions. The unreliable numerical solutions usually appear when the set of parameters is located near the boundary of the stability region of the continuous problem. As a result, the optimal numerical method for long time simulation may be the one whose stability region agrees with that of the original continuous PDEs.

The following problems on the subject remain to be considered in the future:

- long time stability analysis of some classical numerical methods for solving the typical test model (1);
- construction of high-order numerical schemes, whose stability region agrees with that of the original continuous problems;
- extension of the long time stability analysis for the other partial differential equations with delay.

## Figures and Tables

**Table 1:** The numerical solutions at different time for (1) with *b* = 1.01.

*t*	200	400	600	800	1000	1200
CDM	2.04*E* + 1	1.99*E* + 2	1.93*E* + 3	1.87*E* + 4	1.82*E* + 5	1.77*E* + 6
ACFMD	5.70	1.54*E* + 1	4.18*E* + 1	1.33*E* + 2	3.06*E* + 2	8.31*E* + 2
FEM	1.57*E* + 1	1.76	8.80*E* − 1	6.58*E* − 1	4.90*E* − 1	3.69*E* − 1

**Table 2:** The numerical solutions at different time for (1) with *b* = 0.99, *h* = *π*/8, and *k* = 1/20.

*t*	200	400	600	800	1000	1200
CDM	2.77	3.66	4.85	6.41	8.49	1.12*E* + 1
ACDM	7.66*E* − 1	2.81*E* − 2	1.03*E* − 1	3.78*E* − 2	1.38*E* − 2	5.06*E* − 3
FEM	2.10*E* − 1	2.11*E* − 2	2.12*E* − 3	2.14*E* − 4	2.15*E* − 5	2.16*E* − 6

**Table 3:** The numerical solutions at different time for (1) with *b* = 0.99, *h* = *π*/16, and *k* = 1/40.

*t*	200	400	600	800	1000	1200
CDM	1.06	5.34*E* − 1	2.70*E* − 1	1.37*E* − 1	6.917*E* − 2	3.491*E* − 2
ACDM	7.66*E* − 1	2.81*E* − 2	1.03*E* − 1	3.77*E* − 2	1.38*E* − 2	5.06*E* − 3
FEM	5.55*E* − 1	1.47*E* − 1	3.91*E* − 2	1.03*E* − 2	2.76*E* − 3	7.32*E* − 4
